# Co-Immobilization of Laccase and Mediator into Fe-Doped ZIF-8 Significantly Enhances the Degradation of Organic Pollutants

**DOI:** 10.3390/molecules29020307

**Published:** 2024-01-07

**Authors:** Zixuan Li, Qinghong Shi, Xiaoyan Dong, Yan Sun

**Affiliations:** 1 Department of Biochemical Engineering, School of Chemical Engineering and Technology, Tianjin University, Tianjin 300350, China; lzx123@tju.edu.cn (Z.L.); qhshi@tju.edu.cn (Q.S.); d_xy@tju.edu.cn (X.D.); 2 Key Laboratory of Systems Bioengineering and Frontiers Science Center for Synthetic Biology (Ministry of Education), Tianjin University, Tianjin 300350, China

**Keywords:** laccase, mediator, co-immobilization, MOFs, organic pollutants, biodegradation

## Abstract

Co-immobilization of laccase and mediator 2,2′-azino-bis(3-ethylbenzothiazoline-6-sulphonic acid) (ABTS) for wastewater treatment could simultaneously achieve the reusability of laccase and avoid secondary pollution caused by the toxic ABTS. Herein, Fe-induced mineralization was proposed to co-immobilize laccase and ABTS into a metal–organic framework (ZIF-8) within 30 min. Immobilized laccase (Lac@ZIF-8-Fe) prepared at a 1:1 mass ratio of Fe^2+^ to Zn^2+^ exhibited enhanced catalytic efficiency (2.6 times), thermal stability, acid tolerance, and reusability compared to free laccase. ABTS was then co-immobilized to form Lac+ABTS@ZIF-8-Fe (ABTS = 261.7 mg/g). Lac@ZIF-8-Fe exhibited significantly enhanced bisphenol A (BPA) removal performance over free laccase due to the local substrate enrichment effect and improved enzyme stability. Moreover, the Lac+ABTS@ZIF-8-Fe exhibited higher BPA removal efficiency than the free laccase+ABTS system, implying the presence of a proximity effect in Lac+ABTS@ZIF-8-Fe. In the successive malachite green (MG) removal, the MG degradation efficiency by Lac@ZIF-8-Fe was maintained at 96.6% at the fifth reuse with only an extra addition of 0.09 mM ABTS in each cycle. As for Lac+ABTS@ZIF-8-Fe, 58.5% of MG was degraded at the fifth cycle without an extra addition of ABTS. Taken together, this research has provided a novel strategy for the design of a co-immobilized laccase and ABTS system for the degradation of organic pollutants.

## 1. Introduction

Organic contaminants in industrial wastewater, including phenols, organic dyes, and pharmaceuticals, have gained extensive attention due to their substantial risks to human health and environmental safety [1]. As such, different strategies, including physical adsorption, photo-oxidative degradation, advanced oxidation processes, and biological treatment, have been exploited to remove these organic contaminants in industry effluents [2]. Among the above-mentioned methods, biological treatment has attracted increasing attention due to its mild, green, and self-controlled operational process [3,4].

Laccase, a member of the multi-copper oxidase superfamily, shows the ability to oxide a variety of inorganic and organic compounds. Nevertheless, the low redox potential (0.4–0.8 V vs. normal hydrogen electrode (NHE)) of free laccase limits its application when degrading the compounds with high redox potential or those that are not easy to contact with the active center of laccase [5]. Mediators, such as 2,2-azino-bis-3-ethylbenzothiazoline-6-sulfonic acid (ABTS), 1-hydroxybenzotriazole (HBT), and 2,2,6,6-tetramethylpiperidine-1-oxyl (TEMPO), are small molecular compounds with low redox potential, which can promote the oxidation process between laccase and substrate [6]. Therefore, laccase–mediator systems (LMSs) have been widely used to improve the catalytic performance of laccase [7]. Among various LMSs, the laccase–ABTS system performs better on biodegradation of organic pollutants [8]. However, free laccase and mediators are expensive, unstable, and unrecyclable in aqueous solution, thus leading to a high cost. Moreover, the toxic mediators may cause secondary pollution of the environment [7]. Hence, co-immobilization of laccase and mediators to support points out the direction for reasonable and highly efficient usage of LMSs.

Up to now, LMSs have been successfully immobilized onto cellulose microspheres, magnetic chitosan, geopolymer microspheres, and so on [9,10,11]. Gu [9] constructed porous cellulose microspheres modified with polydopamine and polymeric glycidyl methacrylate through a two-step chemical modification, and then ABTS and laccase were co-immobilized onto it through physical encapsulation and covalent linking, respectively. Qiu [10] immobilized ABTS onto magnetic chitosan modified with amino-functionalized ionic liquid and Cu^2+^, while laccase was immobilized through chelation interaction. Recently, Shan [11] optimized the structure of geopolymer microspheres by doping homologous amino acids (His and Cys), aiding in the development of porous structures and reducing mass transfer hindrances. Then, ABTS and laccase were co-immobilized through step-by-step adsorption and used for Congo red decolorization. Noteworthily, evaluations suggest that the above-mentioned strategies might be time-consuming, costly, and/or inefficient. The rational design of immobilization support and method would optimize the co-immobilization process. Recently, metal–organic frameworks (MOFs), a kind of material constructed by metal ions or clusters coordinated to organic ligands, have been extensively studied in the fields of separation, catalysis, environmental remediation, and enzyme immobilization [12,13,14]. Biomimetic mineralization is widely used to in situ embed enzyme into zeolitic imidazolate framework-8 (ZIF-8), a representative MOF formed by Zn^2+^ and 2-methylimidazole (2-Hmim). This is presumably because it can be synthesized under biologically compatible conditions and does little damage to enzyme activity in many cases [15]. However, it often takes a long time (~12 h) to achieve enzyme encapsulation into ZIF-8. Moreover, it is worth noting that ZIF-8 remains stable under neutral or alkaline conditions and rapidly decomposes in an acidic environment, which is needed for laccase catalysis [5].

**Scheme 1:**
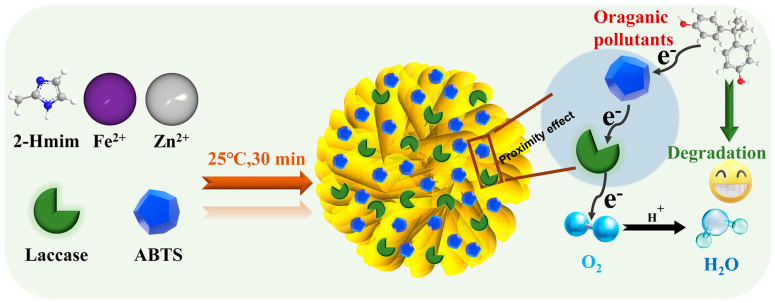
Schematic illustration of facile co-immobilization of laccase and ABTS into Fe-doped ZIF-8 (ZIF-8-Fe) via in situ mineralization.

In this study, we proved that ABTS and laccase could be rapidly co-immobilized into ZIF-8 (Lac+ABTS@ZIF-8). However, Lac+ABTS@ZIF-8 showed an obvious loss of laccase activity and was fairly unstable under an acidic environment. Considering the superior chemical stability of Fe-doped ZIF-8 (ZIF-8-Fe) in the acidic environment and the strong coordination interaction between SO_3_^−^ of ABTS and Fe^3+^ [16,17], we proposed to facilely and environmentally co-immobilize laccase and ABTS into ZIF-8-Fe through a Fe-induced mineralization strategy (Lac+ABTS@ZIF-8-Fe, Scheme 1). The amounts of the introduced iron and laccase concentration for immobilization were first optimized (Lac@ZIF-8-Fe). Afterward, ABTS was co-immobilized with laccase under the optimum conditions. The activity, stability, and reusability of Lac@ZIF-8-Fe were investigated. Furthermore, the removal of typical organic pollutants, such as bisphenol A (BPA) and malachite green (MG), by using free laccase, Lac@ZIF-8-Fe, and Lac+ABTS@ZIF-8-Fe were evaluated systematically to demonstrate the potential of the co-immobilized LMS for the treatment of organic pollutants.

## 2. Results and Discussion

### 2.1. Characteristics of Lac@ZIF-8-Fe and Lac+ABTS@ZIF-8-Fe

**Figure 1:**
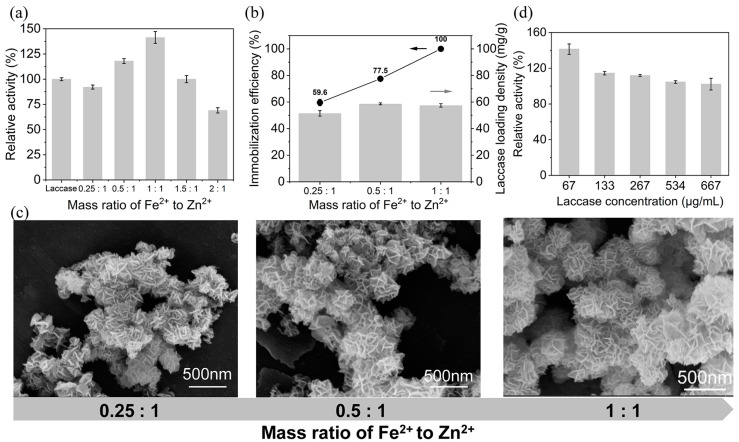
Effect of the mass ratio of Fe^2+^ to Zn^2+^ on the (**a**) relative activity and (**b**) immobilization efficiency and laccase loading density of Lac@ZIF-8-Fe. (**c**) SEM images of the as-prepared Lac@ZIF-8-Fe with different mass ratios (0.25:1 to 1:1) of Fe^2+^ to Zn^2+^. As for (**a**–**c**), the laccase concentration was set as 67 μg/mL. (**d**) Effect of laccase concentration on the relative activity of the as-prepared Lac@ZIF-8-Fe. The activity of free laccase was defined as 100% for comparison (determination of laccase activity: laccase concentration, 2 μg/mL; ABTS concentration, 0.5 mM; pH 6.0; 30 °C).

As shown in Figure S1a, Fe^2+^ could boost the encapsulation of laccase into ZIF-8. With the protonation of 2-Hmim, an alkaline environment was created. Hence, the Fe^2+^ would transform to Fe(OH)_2_, which showed the ability to accelerate the nucleation of ZIF-8 [16]. After a 30-min reaction, the system with the addition of Fe^2+^ turned opaque, and orange/dark green Lac@ZIF-8-Fe was collected. By contrast, the control system without the addition of Fe^2+^ remained transparent, and no precipitate was observed. The relative activity of the as-prepared Lac@ZIF-8-Fe gradually increased to 141% and then decreased to 69% with increasing iron content (Figure 1a). During the mineralization process, Fe^2+^ tended to evolve into chemically stable Fe^3+^, thus resulting in the formation of the orange Lac@ZIF-8-Fe [16]. In contrast, the dark green Lac@ZIF-8-Fe at a Fe^2+^ to Zn^2+^ mass ratio of 1:2 indicated the incomplete transformation of the added Fe^2+^ into Fe^3+^ (Figure S1a). Considering the intrinsic reducibility of Fe^2+^, the declined activity of Lac@ZIF-8-Fe at high Fe^2+^ to Zn^2+^ mass ratios (1.5:1 and 2:1) might be attributed to the presence of residual Fe^2+^ (Figure S1a). As shown in Figure 1b,c, when the mass ratio of Fe^2+^ to Zn^2+^ was between 0.25:1 and 1:1, the as-prepared Lac@ZIF-8-Fe showed similar laccase loading densities (51.5, 58.6, and 57.5 mg/g) and nanoflower-shaped structures. According to the low-resolution SEM image (Figure S1b), it was counted that the as-prepared Lac@ZIF-8-Fe at the Fe^2+^ to Zn^2+^ mass ratio of 1:1 had an average size of approximately 500 nm (Figure S1c). Considering the highest laccase immobilization efficiency (100%) under the condition, the mass ratio of 1:1 was employed in the subsequent experiments. As an acidic enzyme, laccase was negatively charged (pI = 4.2, Table S1 in Supplementary Materials) and tended to induce the formation of ZIF-8-Fe around its surface [18], thus leading to the increasing mass of the formed Lac@ZIF-8-Fe (1.6 to 3.2 mg, Figure S2a) with increasing laccase concentration. However, the relative activity of Lac@ZIF-8-Fe gradually decreased from 141% to 102% with increasing laccase concentration (Figure 1d), but the immobilization efficiency stayed at 100% (Figure S2b). The increased laccase loading density would lead to the crowding effects of the immobilized laccase, thus resulting in gradually reduced activity [19]. In addition, the high-density Lac@ZIF-8-Fe (247.1 and 285.7 mg/g) was assembled with larger and thicker petals (Figure S2c,d), which might further limit the laccase activity due to the higher mass transfer resistance. Based on the above results, the immobilization condition of Fe^2+^ to Zn^2+^ mass ratio at 1:1 and laccase concentration at 67 μg/mL was used for further study.

**Figure 2:**
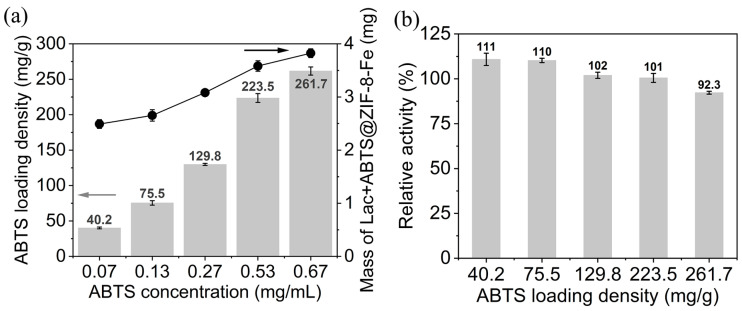
(**a**) Effect of ABTS concentration on the ABTS loading density (mg/g) and mass (mg) of as-prepared Lac+ABTS@ZIF-8-Fe. (**b**) The relative activity of Lac+ABTS@ZIF-8-Fe with different ABTS loading densities. As for (**a**,**b**), the laccase concentration was set as 67 μg/mL. The activity of free laccase was defined as 100% for comparison (determination of laccase activity: laccase concentration, 2 μg/mL; ABTS concentration, 0.5 mM; pH 6.0; 30 °C).

Considering that the SO^3−^ groups of ABTS have the ability to bind Zn^2+^ via an electrostatic interaction [20], it is speculated that ABTS could also accelerate the mineralization process of ZIF-8. As expected, the white precipitate encasing laccase and ABTS (Lac+ABTS@ZIF-8) were rapidly obtained even without iron doping (Figure S3a). Measurements of laccase and ABTS in the supernatant of the immobilization broth demonstrated that both laccase and ABTS were 100% encapsulated. Different from Lac@ZIF-8-Fe, Lac+ABTS@ZIF-8 displayed a mace-shaped structure with a length of 1~2 μm (Figure S3b). However, Lac+ABTS@ZIF-8 only retained 4.4% activity of free laccase (Figure S3c), possibly due to the significant mass transfer resistance caused by the special structure. Subsequently, ABTS was co-immobilized with laccase into ZIF-8-Fe with nearly 100% encapsulation efficiency at different ABTS concentrations (Figure S4). As shown in Figure 2a, the mass of the formed Lac+ABTS@ZIF-8-Fe increased by over two folds with increasing ABTS concentration from 0.07 to 0.67 mg/mL, and the mass of Lac+ABTS@ZIF-8-Fe (2.5 mg) obtained at 0.07 mg/mL ABTS was about 50% more than that of Lac@ZIF-8-Fe (1.6 mg) obtained at the same conditions, which further confirmed that ABTS could accelerate the formation of ZIF-8. The ABTS loading density of Lac+ABTS@ZIF-8-Fe increased from 40.2 to 261.7 mg/g with increasing ABTS concentration. Similar to Lac@ZIF-8-Fe, Lac+ABTS@ZIF-8-Fe also exhibited a nanoflower-shaped structure (Figure S5). The relative activity of Lac+ABTS@ZIF-8-Fe gradually decreased from 111% to 92.3% with the increased ABTS loading density (Figure 2b), possibly due to the excessive amount of ABTS blocking the active site of laccase and inhibiting its activity.

**Figure 3:**
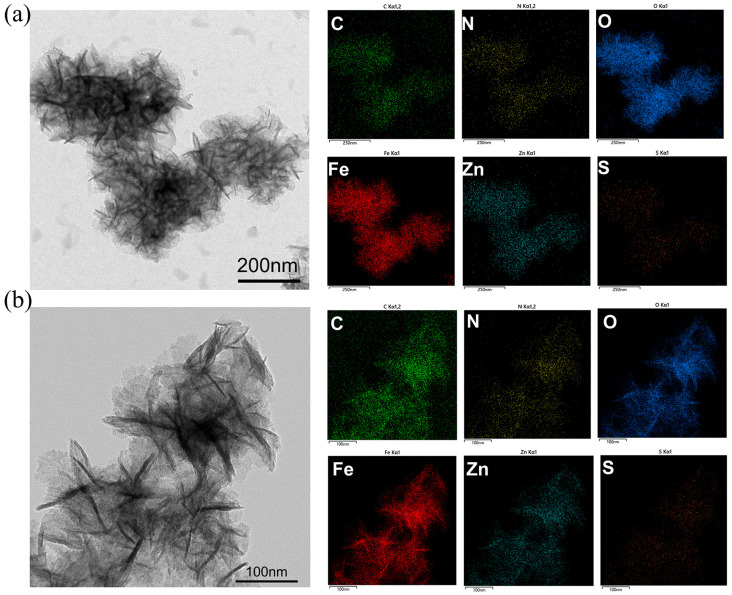
TEM images and EDS analysis of (**a**) Lac@ZIF-8-Fe and (**b**) Lac+ABTS@ZIF-8-Fe.

**Figure 4:**
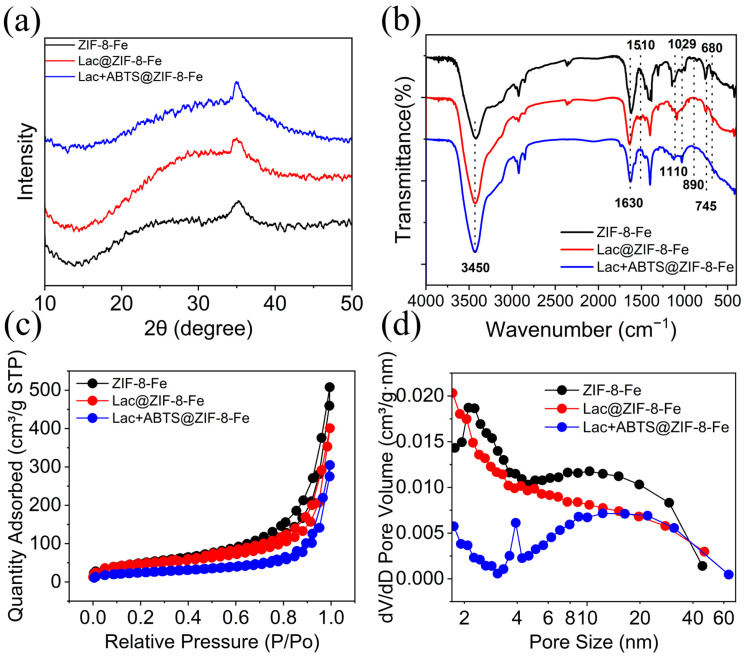
(**a**) XRD patterns, (**b**) FT-IR spectra, (**c**) N_2_ adsorption–desorption isotherms, and (**d**) pore distributions of ZIF-8-Fe, Lac@ZIF-8-Fe, and Lac+ABTS@ZIF-8-Fe.

The TEM images further confirmed the nanoflower-shaped structure of Lac@ZIF-8-Fe and Lac+ABTS@ZIF-8-Fe, which were composed of thin nanosheets (Figure 3). Meanwhile, the nanoparticles (~5 nm) embedded in the nanosheets might be iron oxides formed during the mineralization process of Fe^2+^ (Figure S6), which were conducive to the formation of a mesoporous structure [16]. The EDS images of Lac@ZIF-8-Fe indicated that the elements C, N, O, Fe, Zn, and S were uniformly distributed, and the S element should be attributed to cysteine and methionine of the immobilized laccase. With doping of ABTS, the percentage of S in both samples increased from 0.49% to 1.25% due to sulfonic groups in ABTS. As shown in Figure 4a, all samples exhibited a strong and broad peak at 35°, indicating the presence of an amorphous structure. This might be due to the lower concentrations of 2-Hmim (97 mM) and Zn^2+^ (1.2 mM) used in this study compared to the previous work [21], leading to the incomplete coordination between 2-Hmim and Zn^2+^. As shown in Figure 4b, the characteristic peaks at 3450 and 1630 cm^−1^ were attributed to the hydration water in all samples. The peaks at 1110 and 745 cm^−1^ indicated the formation of Fe-N-C bonds, which were conducive to the structural stability of the samples in an acidic environment [16]. The strong vibration peak at 680 cm^−1^ corresponding to the Fe-O bond further confirmed the existence of iron oxides in all the samples. As for Lac@ZIF-8-Fe and Lac+ABTS@ZIF-8-Fe, a band centered at 1510 cm^−1^ (resulting from the combination of N-H bending mode and C-H stretch mode) appeared and corresponded to the amide II signal of the enzymes [22]. Moreover, the signals at 1029 and 890 cm^−1^ of Lac+ABTS@ZIF-8-Fe are assigned to the stretching and bending modes of the SO^3−^ groups of the ABTS molecule. As shown in Figure 4c, the type-IV hysteresis loops in the P/P_0_ = 0.8–1.0 range indicated that the mesopores were dominant in all the samples. This was further supported by the pore size distribution shown in Figure 4d. As listed in Table S2, the average pore sizes of the samples were 12.0, 10.0, and 12.3 nm, respectively, large enough to ensure the entry and free diffusion of laccase substrates like ABTS (0.64 nm × 0.64 nm × 1.74 nm) in the immobilized enzyme preparations [5]. The specific surface areas of ZIF-8-Fe, Lac@ZIF-8-Fe, and Lac+ABTS@ZIF-8-Fe were 175.6, 161.8, and 89.8 m^2^/g, respectively. The gradually decreased specific surface area could be attributed to the encapsulated laccase and ABTS, which could partially occupy and/or block the pore spaces of ZIF-8-Fe.

### 2.2. Enzymatic Properties of Free Laccase and Lac@ZIF-8-Fe

**Figure 5:**
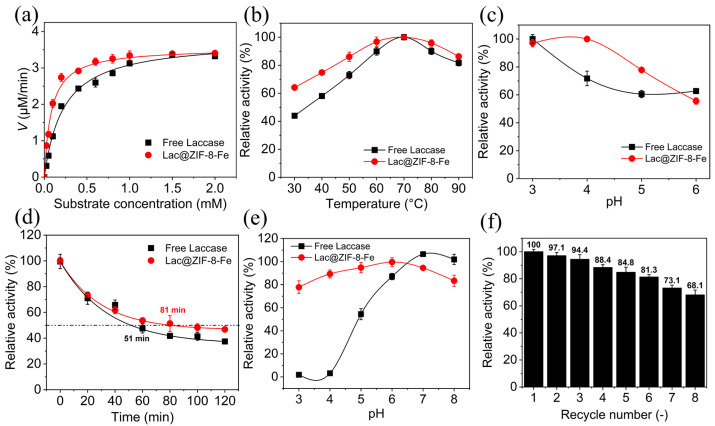
(**a**) Kinetics curve of ABTS oxidation catalyzed by laccase and Lac@ZIF-8-Fe. Effects of (**b**) temperature (at pH 6.0) and (**c**) pH (at 30 °C) on the activities of free and immobilized laccase. (**d**) Thermal stabilities (2 h incubation at 80 °C and pH 6.0) and (**e**) pH tolerances (2 h incubation at 30 °C and each pH) of free and immobilized laccase. (**f**) Reusability of Lac@ZIF-8-Fe to oxide 0.5 mM ABTS. The solid lines in (**d**) were calculated by fitting the deactivation data to the simplified series deactivation model (Equation (1)).

To investigate the catalytic performance of laccase and Lac@ZIF-8-Fe, the kinetic parameters of the two enzyme preparations were determined through a steady-state kinetic assay (Figure 5a), and the kinetic data were fitted. As listed in Table 1, the *K*_m_ value of Lac@ZIF-8-Fe (0.08 mM) was approximately one-third that of free laccase (0.22 mM), indicating an enhanced affinity of Lac@ZIF-8-Fe for ABTS. Considering the opposite charges of ABTS and Lac@ZIF-8-Fe (Table S1), the increased affinity might be attributed to the local enrichment of ABTS via electrostatic interactions. In addition, the similar *k*_cat_ values of the two enzyme preparations indicated that laccase activity was kept in the MOF support. Thus, due to the significantly decreased *K*_m_, the catalytic efficiency, *k*_cat_/*K*_m_, of Lac@ZIF-8-Fe was 2.6 times that of free laccase. However, it should be pointed out that when laccase was encased into pristine ZIF-8 with a 0.34 nm pore window, its catalytic efficiency was only half of the free laccase due to the steric hindrance of the tight solid framework [23].

Temperature and pH have crucial effects on enzyme activity. As shown in Figure 5b, the activities of free and immobilized laccase initially increased and then decreased with increasing temperature. Both samples showed an optimum temperature of 70 °C. Moreover, compared with free laccase, Lac@ZIF-8-Fe exhibited a broader temperature profile, possibly due to the protective effect of the carrier, which enhanced the structural rigidity of the laccase [24]. As an acidophilic enzyme, the activity of free laccase decreased from 100% to 62.7% with increasing pH from 3.0 to 6.0 (Figure 5c). In contrast, the optimum pH value of Lac@ZIF-8-Fe shifted from 3.0 to 4.0, which could be attributed to the ionic interaction between the laccase and the carriers [25].

Given the inherent stability of bacterial laccase, the thermal stabilities of both enzyme preparations were evaluated at a high temperature (80 °C) (Figure 5d). It was found that the deactivation process of the enzyme preparations matched well with the simplified series-deactivation model [26].

(1) $$A=(1-a)e^{-kt}+a$$

where *A* is the residual enzyme activity; a is the ratio of specific activity of the denatured state (*E*_1_) to that of the native state (*E*_0_); *k* (min^−1^) is the deactivation rate constant for the transformation of *E*_0_ to *E*_1_, and t is incubation time (min). Thermal deactivation parameters were obtained by fitting the data to Equation (1) and listed in Table S3. The half-life time of Lac@ZIF-8-Fe (81 min) was 1.6 times that of free laccase (51 min), indicating improved thermal stability after immobilization. As depicted in Figure 5e, the acid tolerance of Lac@ZIF-8-Fe was superior to that of free laccase. For instance, the free laccase lost 92.1% and 96.8% of its initial activity after a 2-hour incubation at pH 3.0 and 4.0, respectively. In contrast, Lac@ZIF-8-Fe maintained 77.7% and 89.3% of its initial activity after incubation at pH 3.0 and 4.0, respectively. Considering the electropositivity of the imidazole ring of 2-Hmim, an alkaline microenvironment might be created around the immobilized laccase. This alkaline microenvironment may counteract the influence of H^+^ in the solution [10]. As reported previously, the interaction between OH^−^ and the copper ions of laccase could potentially impede the flow of electrons, consequently resulting in a decline in the enzyme’s activity [27]. Hence, the stability of Lac@ZIF-8-Fe was found to be inferior to that of free laccase when exposed to a weak alkaline environment (pH 7.0 to 8.0).

Reusability is a significant advantage of immobilized biocatalysts in industry applications. As shown in Figure 5f, Lac@ZIF-8-Fe showed excellent reusability, with retention of over 68.1% in relative activity after eight cycles. The decrease in activity could be attributed to the dissociation and deactivation of laccase molecules during the consecutive recycling test. Even so, Lac@ZIF-8-Fe demonstrated superior reusability compared to laccase encapsulated in pristine ZIF-8, which retained only 48% of its initial activity after seven cycles [23].

### 2.3. BPA Removal Experiments

**Figure 6:**
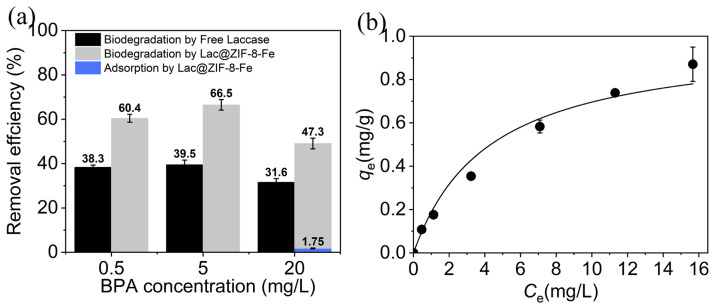
(**a**) BPA removal efficiencies with laccase and Lac@ZIF-8-Fe at different BPA concentrations at 50 °C. Enzyme concentration, 10 μg/mL; reaction time, 12 h. (**b**) Adsorption isotherm of BPA onto ZIF-8-Fe (5 mg/mL) at 50 °C and pH 6.0 (equilibrium data were obtained by 12 h adsorption).

To enhance the BPA degradation performance of laccase, the impacts of temperature (30–80 °C) and pH (3.0–7.0) were investigated (Figure S7). Although the highest removal efficiency was achieved at 70 °C, considering the declined thermal stability of enzyme molecules at high temperatures (Figure 5d), a temperature of 50 °C was selected for BPA degradation (Figure S7a). As shown in Figure S7b, pH 6.0 was most suitable for laccase to remove BPA. The suboptimal removal efficiency in an acidic medium (pH < 6.0) could be attributed to the comparatively low acid tolerance of laccase, as shown in Figure 5e. As shown in Figure 6a, the BPA removal efficiencies of free laccase were 38.3% and 39.5% at low BPA concentrations of 0.5 and 5 mg/L, respectively. As reported previously, during the process of enzymatic degradation, BPA could be oxidized into radicals. These radicals possess the potential to initiate the formation of enzyme–radical conjugates, resulting in a decrease in enzyme activity [28]. Hence, a diminished BPA removal efficiency (31.6%) was observed when BPA concentration reached a high level of 20 mg/L. It was noteworthy that Lac@ZIF-8-Fe exhibited superior BPA removal ability compared to free laccase across the concentration range (0.5–20 mg/L). After a 12-h reaction, about 60.4%, 66.5%, and 47.3% of BPA were removed at 0.5, 5.0, and 20 mg/mL, respectively.

Of the BPA removed by Lac@ZIF-8-Fe at a concentration of 20 mg/L, part might be attributed to the adsorption of BPA molecules to the carrier (ZIF-8-Fe). Thus, the BPA adsorption isotherm of ZIF-8-Fe was determined. As shown in Figure 6b, the adsorption equilibrium exhibited a good fit to the Langmuir isotherm given below.

(2) $$q_{e}=\frac{q_{m}C_{e}}{K_{d}+C_{e}}$$

where *q*_m_ is the adsorption capacity; *K*_d_ is the dissociation constant; *q*_e_ is the equilibrium adsorption density, and *C*_e_ is the equilibrium BPA concentration in the supernatant after adsorption. The values of *q*_m_ and *K*_d_ were determined to be 0.99 mg/g and 4.17 mg/L, respectively. It was calculated that about 1.75% BPA was adsorbed onto Lac@ZIF-8-Fe after the reaction (Figure 6a). Considering the molecular structures of BPA and ZIF-8-Fe, it was inferred that the adsorption was attributed to multi-binding affinities, such as π-π interaction, hydrogen bonding, coordination interaction, and electrostatic interactions (Figure S8). However, a too-strong interaction between BPA and the immobilization support would limit the substrate binding to the enzyme [25], thus reducing the BPA degradation ability of laccase. Herein, the large *K*_d_ value suggests reversible adsorption between BPA and ZIF-8-Fe, resulting in the local enrichment of BPA, which then promotes BPA degradation by Lac@ZIF-8-Fe (Figure 6a). Furthermore, the enhanced stability of Lac@ZIF-8-Fe might be another factor for the better BPA removal ability (Figure 5d,e).

**Figure 7:**
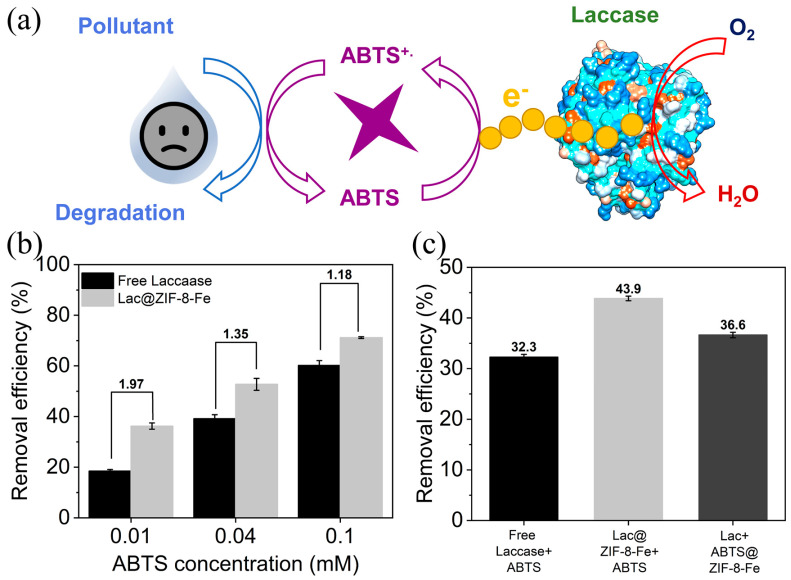
(**a**) Typical catalytic mechanism of the laccase–mediator system. (**b**) BPA removal efficiencies of free laccase and Lac@ZIF-8-Fe with different ABTS concentrations at 50 °C. Enzyme concentration, 2 μg/mL; reaction time, 12 h; BPA concentration, 20 mg/L. (**c**) BPA removal efficiencies of free laccase+ABTS, Lac@ZIF-8-Fe+ABTS, and Lac+ABTS@ZIF-8-Fe at 50 °C. Enzyme concentration, 2 μg/mL; reaction time, 12 h; BPA concentration, 20 mg/L; ABTS kept an equivalent concentration of 0.036 mM in all three reaction systems.

Then, the LMSs were evaluated for BPA removal. Figure 7a shows the mechanism of LMSs for dramatically increasing the catalytic performance of laccase. In the LMS-based degradation, the mediator (ABTS) is oxidized into a high-potential intermediate (ABTS^+•^) by laccase. Meanwhile, the electrons originating from ABTS oxidation are transferred to oxygen, resulting in its reduction to water. ABTS^+•^ has much higher efficiency in oxidizing organic pollutants than laccase and is then reduced to its initial form and participates in a subsequent redox cycle [7]. As shown in Figure 7a, with the assistance of 0.1 mM ABTS, 2 μg/mL Lac@ZIF-8-Fe showed higher BPA removal efficiency (60.2%) than 10 μg/mL of the Lac@ZIF-8-Fe-only system (47.3%, Figure 6a). However, considering the toxicity of ABTS, its application in minimal quantities is desired to avoid secondary pollution. As shown in Figure 7b, both free laccase and Lac@ZIF-8-Fe showed gradually improved removal efficiencies with increasing ABTS concentration. The removal efficiencies of Lac@ZIF-8-Fe were 1.97-, 1.35-, and 1.18-fold higher than those of free laccase at ABTS concentrations of 0.01, 0.04, and 0.1 mM, respectively. The high efficiency of Lac@ZIF-8-Fe might be attributed to the enhanced affinity of Lac@ZIF-8-Fe to ABTS (Figure 5a) and is conducive to its practical application.

Next, the co-immobilized laccase and ABTS system was investigated. As shown in Figure S9, the BPA removal efficiencies of Lac+ABTS@ZIF-8-Fe gradually reached a plateau (from 34.0% to 71.6%) with increasing ABTS loading density from 40.2 to 261.7 mg/g. This indicates that the ABTS encapsulated in Lac+ABTS@ZIF-8-Fe (261.7 mg/g) might be enough for laccase to degrade BPA. Therefore, the Lac+ABTS@ZIF-8-Fe with the ABTS loading density of 267.1 mg/g was selected for further study. Among the three catalytic systems at the same laccase and ABTS concentrations (Figure 7c), Lac@ZIF-8-Fe+ABTS exhibited the best BPA removal efficiency (43.9%), followed by Lac+ABTS@ZIF-8-Fe (36.6%), while the free LMS showed the lowest BPA removal efficiency (32.3%). Compared with Lac+ABTS@ZIF-8-Fe, the higher BPA removal efficiency of Lac@ZIF-8-Fe+ABTS could be attributed to its higher laccase activity (Figure 1d and Figure 2b) and lower mass transfer resistance between BPA molecules and free ABTS (Figure 4c). Although Lac+ABTS@ZIF-8-Fe was not superior over Lac@ZIF-8-Fe+ABTS in degrading BPA in a single batch, its advantage was in its reusability of both laccase and ABTS to be discussed in the following section. In addition, the lowest removal efficiency of the free laccase+ABTS system indicated the existence of a proximity effect between laccase and ABTS in Lac+ABTS@ZIF-8-Fe, which would shorten the mass transfer distance and thus accelerate the degradation process [29]. Compared with the reported co-immobilized LMSs in the literature [5,9,10,11,30,31], the present Lac+ABTS@ZIF-8-Fe system exhibited considerable advantages (Table S4). Firstly, in the preparations, Lac+ABTS@ZIF-8-Fe was fabricated via a one-pot strategy under mild conditions within 30 min, while the preparation of other catalysts needs two/three steps and a long time (7.5–96 h). Secondly, the literature methods involved the use of more raw materials such as organic solvents, strong acid or alkaline, and toxic reagents, which would inevitably lead to a high cost and environmental pollution, compromising the principle of “Green chemistry” of biodegradation. Thirdly, as for catalytic activity, Lac+ABTS@ZIF-8-Fe performed better than most of the co-immobilized systems that showed lower activity than the free laccase+ABTS system [5,30]. This could be attributed to the high activity recovery of Lac+ABTS@ZIF-8-Fe, which illustrated the good compatibility of the immobilization process to laccase. Taken together, the Fe-induced co-immobilization of LMS in ZIF-8 is of great potential for environmental remediation.

### 2.4. Reusability of Immobilized Laccase for MG Removal

**Figure 8:**
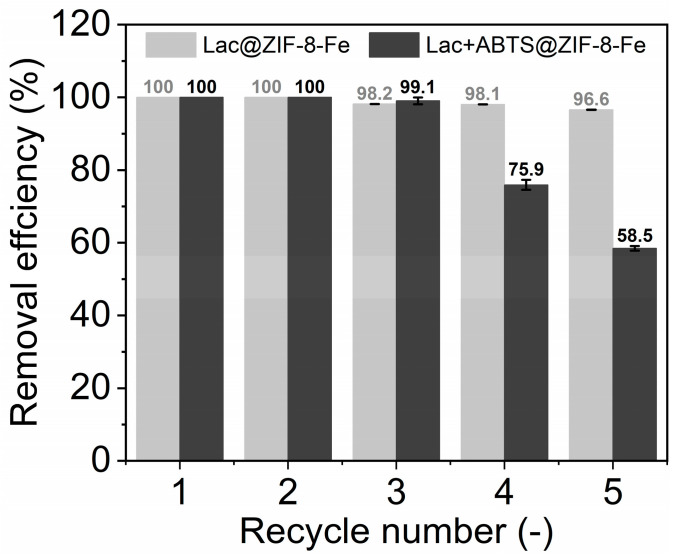
Reusability of Lac@ZIF-8-Fe with extra addition of 0.09 mM ABTS and Lac+ABTS@ZIF-8-Fe for MG removal. MG concentration, 10 mg/L; enzyme concentration, 10 μg/mL; temperature, 50 °C; reaction time, 1 h.

To explore the universality of this immobilization method for the removal of organic pollutants, MG was used to determine the reusability of Lac@ZIF-8-Fe and Lac+ABTS@ZIF-8-Fe. As shown in Figure 8, Lac@ZIF-8-Fe maintained about 96.6% of its initial activity after five cycles, which was significantly higher than those of laccase immobilized onto a magnetic carbon carrier and a regular MOF (NH_2_-MIL-53(Al)), which showed only 80% and 85% removal efficiencies at the fifth cycle, respectively [32,33]. More importantly, to maintain the high organic pollutant removal efficiency during the successive cycling, a high concentration of ABTS (0.2–0.5 mM) was used in most research [32,34,35]. In contrast, only 0.09 mM ABTS was used in this study due to the high affinity of ZIF-8-Fe to ABTS (Figure 5a). It was speculated that the slightly decreased activity during the cycling might be attributed to the partial loss of immobilized laccase during the recycling process.

However, the reusability of Lac+ABTS@ZIF-8-Fe was slightly less than Lac@ZIF-8-Fe (Figure 8). The MG removal efficiency dropped from 100% to 58.5% after five cycles. On the one hand, as the pore size of the carrier (12.3 nm) was larger than that of ABTS, part of the fixed ABTS leaked off during the recycling, leading to reduced activity. After three cycles, about 61.4% of the immobilized ABTS was retained (Figure S10a). On the other hand, the slightly poorer enzyme stability of Lac+ABTS@ZIF-8-Fe might be another factor. As shown in Figure S10b, after a 3-hour incubation at 50 °C, the residual activity of Lac+ABTS@ZIF-8-Fe was 19.2% lower than that of Lac@ZIF-8-Fe. This implies that the co-immobilized ABTS in Lac+ABTS@ZIF-8-Fe would readily react with laccase full of active amino acid residues, leading to the faster denaturation and inactivation of laccase molecules [36]. According to previous reports (Table S4), it is likely that the co-immobilized LMSs with ABTS encapsulated in the core while laccase immobilized on the surface exhibited better reusability [9,10,31]. Therefore, further research into the prevention of ABTS leakage and compartmentalized immobilization of ABTS and laccase would address the above demerit of Lac+ABTS@ZIF-8-Fe.

## 3. Materials and Methods

### 3.1. Chemicals and Materials

BPA (99%), MG (99%), 2-Hmim (98%), FeSO_4_·7H_2_O (99%), zinc acetate dihydrate (Zn (CH_3_COO)_2_·2H_2_O, 98%), acetic acid (99%), sodium acetate anhydrous (analytical grade, AR), methanol (99.9%), and ABTS (98%) were obtained from Aladdin (Shanghai, China). Bovine serum albumin (BSA, 95%) was from Sigma-Aldrich (St. Louis, MO, USA). Coomassie brilliant blue G-250 (AR) was purchased from Dingguo Biotech (Beijing, China). Other reagents of analytical grade were from Yuanli Chemical Technology (Tianjin, China). *Escherichia coli* (*E. coil*) strain containing the laccase gene (CotA gene from *Bacillus subtillis*) was kindly provided by the Qi group and used for laccase expression and production according to the procedure reported previously [32].

### 3.2. Preparation of ZIF-8-Fe, Lac@ZIF-8-Fe, and Lac+ABTS@ZIF-8-Fe

The synthesis of ZIF-8-Fe was based on the previous report with minor modifications [37]. Typically, 4 mg of FeSO_4_.7H_2_O was dispersed into 1 mL of sodium acetate buffer (50 mM, pH 6.0) containing 2-Hmim (11.96 mg). Subsequently, 0.5 mL of Zn (CH_3_COO)_2_·2H_2_O (8 mg/mL) was added to the mixture in which the mass ratio of FeSO_4_.7H_2_O to Zn (CH_3_COO)_2_·2H_2_O (denoted as Fe^2+^ to Zn^2+^) was 1:1. The resulting reactant solution was incubated at 25 °C and shaken at 150 rpm for 30 min; then, the products were collected by centrifugation (9000 rpm, 5 min) and washed three times with sodium acetate buffer (50 mM, pH 6.0). The final orange solid precipitate was lyophilized for further experiments.

Laccase was in situ encapsulated into ZIF-8-Fe via a de novo strategy. The effects of iron content and laccase concentration on the immobilization efficiency, loading density, and activity of Lac@ZIF-8-Fe were explored. Different amounts of FeSO_4_.7H_2_O (1, 2, and 4 mg) were added to 1 mL of laccase solution (100 μg/mL) containing 2-Hmim (11.96 mg). Afterward, 0.5 mL of Zn (CH_3_COO)_2_·2H_2_O (8 mg/mL) was added to the mixture, wherein the mass ratios of Fe^2+^ to Zn^2+^ were 0.25:1, 0.5:1, and 1:1. The reaction and collecting conditions were the same as in the ZIF-8-Fe preparation. To optimize laccase concentration (67–667 μg/mL) for immobilization, a series of Lac@ZIF-8-Fe was synthesized with the mass ratio of Fe^2+^ to Zn^2+^ maintained at 1:1. The immobilization efficiency was defined as the percentage of the enzyme loaded into the support relative to that added into the immobilization system, and the loading density was calculated by mass balance. Bradford method was used to determine the enzyme concentration with BSA as a standard [38].

Laccase and ABTS were co-encapsulated into ZIF-8-Fe through a one-pot method. The effect of ABTS concentration (0.07–0.67 mg/mL) was first explored. Different dosages of ABTS (0.1, 0.2, 0.4, 0.8, and 1.0 mg) were dispersed into 1 mL of laccase solution (100 μg/mL) containing FeSO_4_.7H_2_O (4 mg) and 2-Hmim (11.96 mg), and then 0.5 mL of Zn (CH_3_COO)_2_·2H_2_O (8 mg/mL) was added to the mixture. The prepared Lac+ABTS@ZIF-8-Fe was collected and washed using the above-mentioned methods. The unencapsulated amount of ABTS was determined through GB/T 39100-2020, as reported previously [5].

### 3.3. Characterization of Materials

The morphology of the samples was observed on scanning electron microscopy (SEM) (Apreo S LoVac, FEI, Hillsboro, Oregon, USA) and transmission electron microscopy (TEM) (JEM-2100F, JEOL, Japan). The energy-dispersive X-ray spectroscopy (EDS) was used to analyze the element distribution in samples. The zeta potential of samples was measured on a Malvern Zetasizer instrument (Nano ZS, Malvern Instruments, Malvern, UK). X-ray diffraction (XRD) patterns were recorded on a D8 advance X-ray powder diffractometer (Burker, Germany) with a 2*θ* angle in the range of 10–50° to investigate the crystal structure of all samples. Fourier transform infrared (FT-IR) spectra were measured in the 4000–500 cm^−1^ on an FT-IR spectrometer (Nicolet AVATAR 360, Madison, Wisconsin, USA). The UV-vis-NIR spectra were recorded on a Lambda 35 spectrometer (Perkin Elmer, Waltham, Massachusetts, USA). Nitrogen adsorption/desorption isotherms were measured with ASAP 2420 (Micromeritics, Norcross, Georgia, USA). The specific surface area and pore size distribution of samples were calculated based on the Brunauer–Emmett–Teller (BET) method and the Barret–Joyner–Hallenda model, respectively [39].

### 3.4. Characterization of Enzymatic Properties

Free and immobilized laccase activities were determined by a colorimetric method with ABTS as the substrate. As a common laccase substrate, ABTS could be oxidized into ABTS^+•^, which exhibited absorbance at 420 nm (ε = 3.6 × 10^4^/M/cm). Briefly, 100 μL free or immobilized laccase (20 μg laccase/mL) solution was added into 900 μL sodium acetate buffer (50 mM, pH 6.0) containing 0.5 mM ABTS and then reacted in a water bath at 30 °C for 5 min. After the reaction, the absorbance of the solution was measured via a spectrometer. One unit (U) of laccase was defined as the amount of enzyme that oxidizes 1.0 μM ABTS per minute. To determine the enzymatic reaction kinetics, the initial enzymatic reaction rates of the free and immobilized laccase at different ABTS concentrations (0.5 to 2 mM) were measured at 30 °C and pH 6.0, and the kinetic parameters were obtained according to the Michaelis–Menten equation

(3) $$v=\frac{V_{\max}\left[S\right]}{K_{m}+[S]}$$

where *v* is the reaction rate (μM/min); [S] is the substrate concentration (mM); *V*_max_ is the maximum rate (μM/min), and *K*_m_ is the Michaelis constant (mM).

The effects of temperature (30–90 °C) and pH (3.0–6.0) on free and immobilized laccase activities were studied, and the reaction conditions were the same as the above method. The maximum activity obtained was defined as 100% for comparison.

To explore thermal stability, free or immobilized laccase was continuously incubated in a water bath at 80 °C and pH 6.0, and their residual activities were measured every 20 min. The pH tolerances of free and immobilized laccase were explored in buffers in the pH range of 3.0–8.0 at 30 °C for 2 h. For the above-mentioned experiments, the initial activity of laccases was defined as 100%.

The reusability of Lac@ZIF-8-Fe was explored by measuring its relative activity over 8 cycles using ABTS as the substrate. After each cycle, the Lac@ZIF-8-Fe was centrifuged and washed and then redispersed into a fresh ABTS solution (0.5 mM) for the next run. The activity of the initial cycle was defined as 100%.

### 3.5. Removal of BPA and MG

BPA adsorption experiments were carried out to evaluate the adsorption performance of ZIF-8-Fe to BPA. Briefly, 5 mg/mL ZIF-8-Fe was dispersed into a BPA solution (pH 6.0) of different concentrations (1–20 mg/L), and then the mixture was incubated at 50 °C. After a 12-hour incubation, samples were taken out, and the concentration of residual BPA was determined using a high-performance liquid chromatography (HPLC) system (Shimadzu, Japan) equipped with a C18 column (Welch Ultimate 4.6 × 250 mm XB-C18). BPA detection conditions were as follows: mobile phase, the mixture of menthol and water with a volume ratio of 80:20; flow rate, 0.7 mL/min; detection wavelength, 278 nm; and column temperature, 25 °C. The retention time of BPA under the conditions was 7.0 min.

To evaluate the BPA removal abilities of free laccase and Lac@ZIF-8-Fe, each biocatalyst (10 μg laccase/mL) was incubated with BPA solutions (pH 6.0) of different concentrations at 50 °C for 12 h. The removal efficiency was calculated according to the concentration of BPA before and after the reaction. To determine the amount of BPA adsorbed onto Lac@ZIF-8-Fe, Lac@ZIF-8-Fe was collected, redispersed into methanol, and treated by ultrasonic treatment for 2 h to desorb any bound BPA molecule.

To improve the BPA removal ability of laccase, an electro-mediator (ABTS) was introduced into the reaction system to mediate the oxidation process of BPA. Free laccase or Lac@ZIF-8-Fe (2 μg laccase/mL) was incubated with BPA solution (20 mg/L) with different ABTS concentrations at 50 °C and pH 6.0. After reacting for 12 h, the residual amount of BPA was measured.

To assess the effect of co-immobilization of LMS on the BPA removal, three systems, laccase+ABTS, Lac@ZIF-8-Fe+ABTS, and Lac+ABTS@ZIF-8-Fe, were applied to remove BPA. Briefly, the three systems (2 μg laccase/mL in each system) were incubated with BPA solution (20 mg/L) at 50 °C and pH 6.0. It was worth noting that free laccase and Lac@ZIF-8-Fe systems needed an extra addition of an equal amount of ABTS to that in the Lac+ABTS@ZIF-8-Fe system.

To evaluate the reusability of Lac@ZIF-8-Fe for organic pollutant removal, Lac@ZIF-8-Fe (10 μg laccase/mL) was used for successive MG removal (10 mg/L). In each batch, the reaction system was added with 0.09 mM ABTS and incubated at 50 °C for 1 h. After the reaction, Lac@ZIF-8-Fe was recovered by centrifuging at 4 °C (10,000 rpm, 5 min) and washed for the next batch. In the case of Lac+ABTS@ZIF-8-Fe, it (10 μg laccase/mL) was directly used to remove MG (10 mg/L) for 5 cycles. The reaction conditions were the same as above.

## 4. Conclusions

In this work, a facile one-pot strategy, Fe-induced mineralization, was proposed to co-immobilize laccase and mediator within 30 min. Compared to previously reported strategies [5,9,10,11,30,31], this method was time-saving and had high efficiency and low cost. Under the optimum conditions (Fe^2+^ to Zn^2+^ = 1:1), the immobilized laccase (Lac@ZIF-8-Fe) showed a 1.6-fold increase in catalytic efficiency, *k*_cat_/*K*_m_, over free laccase. Moreover, Lac@ZIF-8-Fe exhibited excellent thermal stability, acid tolerance, and reusability. After the doping of ABTS, the ABTS loading density of Lac+ABTS@ZIF-8-Fe could reach 261.7 mg/g. In BPA degradation, the BPA removal efficiency of Lac@ZIF-8-Fe was 1.5–1.7 times that of free laccase at different BPA concentrations. In addition, even with the addition of ABTS, Lac@ZIF-8-Fe still exhibited better BPA removal ability than free laccase, possibly due to the improved affinity between immobilized laccase and ABTS. The co-immobilized system, Lac+ABTS@ZIF-8-Fe, was also superior in BPA removal over the free LMS due to the proximity effect. Furthermore, Lac@ZIF-8-Fe had excellent reusability in MG removal, with 96.6% removal efficiency retained after five cycles. Though the slightly poor reusability of Lac+ABTS@ZIF-8-Fe was found due to the partial release of ABTS and the deactivation of laccase, the strategy proposed herein was of great potential toward the design and application of co-immobilized laccase–mediator systems.

## Figures and Tables

**Table 1:** Kinetic parameters of the enzyme preparations.

Kinetic Parameters	Free Laccase	Lac@ZIF-8-Fe
*V*_max_ (μM·min^−1^)	3.78	3.55
*k*_cat_ (min^−1^)	119.3	112.4
*K*_m_ (mM)	0.22	0.08
*k*_cat_/*K*_m_(min^−1^·mM^−1^)	542.4	1404

## Data Availability

Data are available upon request from the authors.

## Supplementary Materials

The following supporting information can be downloaded at https://www.mdpi.com/article/10.3390/molecules29020307/s1. Table S1: Zeta potentials of various materials used in this study; Table S2: BET specific surface area and average pore size; Table S3: Thermal deactivation parameters of free and immobilized laccase; Table S4: Comparison of co-immobilized LMSs reported in this work and the literature data; Figure S1: Digital and SEM images and size distribution histograms of the as-prepared Lac@ZIF-8-Fe; Figure S2: Effect of laccase concentration on the mass of the immobilized enzyme preparations, laccase loading density, immobilization efficiency, and morphology of Lac@ZIF-8-Fe; Figure S3: Synthesis and activity characterization of Lac+ABTS@ZIF-8; Figure S4: Effect of ABTS concentration on its encapsulation efficiency; Figure S5: SEM images of the as-prepared Lac+ABTS@ZIF-8-Fe with different ABTS loading densities; Figure S6: High-resolution TEM image of a nanosheet of Lac@ZIF-8-Fe; Figure S7: Effects of temperature and pH on free laccase for BPA removal; Figure S8: Possible mechanism for BPA adsorption onto ZIF-8-Fe; Figure S9: Effect of ABTS loading density of Lac+ABTS@ZIF-8-Fe on BPA removal efficiency at 50 °C; Figure S10: ABTS residual amount of Lac+ABTS@ZIF-8-Fe after each cycle, and thermal stability of Lac@ZIF-8-Fe and Lac+ABTS@ZIF-8-Fe at 50 °C.
