# Supplementary material for: Co-Immobilization of Laccase and Mediator into Fe-Doped ZIF-8 Significantly Enhances the Degradation of Organic Pollutants
Source: Molecules. 2024 Jan 7;29(2):307. doi: 10.3390/molecules29020307 (PMC10819759; doi:10.3390/molecules29020307)
Supplement: Supplementary file 1 [file molecules-29-00307-s001.zip › molecules-2802537-supplementary.pdf]

Co-Immobilization of Laccase and Mediator into Fe-doped ZIF-8 Significantly Enhances the Degradation of Organic Pollutants

Zixuan Li [1], Qinghong Shi [1,2], Xiaoyan Dong [1,2] and Yan Sun [1,2,*]

[1] Department of Biochemical Engineering, School of Chemical Engineering and Technology, Tianjin University, Tianjin 300350, China; lzx123@tju.edu.cn (Z.L.); qhshi@tju.edu.cn (Q.S.); d_xy@tju.edu.cn (X.D.)

[2] Key Laboratory of Systems Bioengineering and Frontiers Science Center for Synthetic Biology (Ministry of Education), Tianjin University, Tianjin 300350, China

* Correspondence: ysun@tju.edu.cn

1. Supporting Tables

Table S1. Zeta potentials of various materials used in this study.

	Laccase	ABTS	ZIF-8-Fe	Lac@ZIF-8-Fe [a]	Lac+ABTS @ZIF-8-Fe [b]
Zeta potential (mV)	-10.1	-28.7	16.7	12.7	6.25

[a] The loading density of laccase was 57.5 mg/g.

[b] The loading densities of laccase and ABTS were 26.2 mg/g and 261.7 mg/g, respectively.

Table S2. BET specific surface area and average pore size.

Samples	Specific surface area (m^2/g)	Average pore size (nm)
ZIF-8-Fe	175.6	12.0
Lac@ZIF-8-Fe	161.8	10.0
Lac+ABTS@ZIF-8-Fe	89.8	12.3

Table S3. Thermal deactivation parameters of free and immobilized laccase.

Parameters	Free laccase	Lac@ZIF-8-Fe
k (min^{-1})	0.029	0.033
a	0.35	0.46
Half-time (min)	51	81

Table S4. Comparison of co-immobilized LMSs reported in this work and literature data.

Reference	Laccase and/or mediator immobilization carriers	Total preparation procedure and time	Catalysts	Targeted pollutant	Degradation time	Removal efficiency	Reusability
**(Liu et al., 2017)**	MIL-100(Fe)	Two steps 96 h	Free laccase<br>Free laccase +ABTS<br>Laccase+ ABTS@MIL-100(Fe)	Indigo carmine	50 min<br>10 min<br>50 min	5%<br>94%<br>95%	-
**(Gu et al., 2019)**	Cellulose beads functionalized with polydopamine and polymeric glycidyl methacrylate	Two steps 33 h	Free laccase<br>Free laccase+ABTS<br>PD-GMA-Ce/ABTS@Lac	Indole	144 h<br>-<br>18 h	1.7%<br>-<br>99.7%	86.3% at the 10th cycle
**(Xue et al., 2020)**	Calcium alginate beads functionalized with polydopamine and polymeric glycidyl methacrylate	Two steps 34 h	Free laccase<br>Free laccase+ABTS<br>Lac/PD-GMA-Ca@ABTS	Acridine	8 h<br>-<br>8 h	1.2%<br>-<br>100%	93.4% at the 4th cycle
**(Qiu et al., 2021)**	Magnetic chitosan functionalized with amino-modified ionic liquid	Three steps 68 h 50 min	Free laccase<br>Free laccase+ABTS<br>MACS-NIL-Cu-lac	2,4-dichlorophenol	12 h<br>-<br>4 h	100%<br>-<br>100%	93.2% at the 6th cycle
**(Shan et al., 2022)**	Geopolymer microspheres synthesized with amino acid (His and Cys)	Two steps 7 h 30 min	Free laccase<br>Free laccase+ABTS<br>Lac-ABTS@GM-H_2C_1	Congo red	28 h	58.1%<br>79.2%<br>94.8%	50.7% at the 10th cycle
**(Lou et al., 2023)**	UiO-66(Zr)-NH_2 grown on polyethylene terephthalate	Three steps 39 h 50 min	Free laccase<br>Free laccase+ABTS<br>PET/ABTS@UiO-66(Zr)–NH_2/Lac	Crystal violet	12 h	18.1%<br>82.9%<br>65%	6.91% at the 4th cycle

**This work**	Iron-doped ZIF-8	One-pot<br>30 min	Free laccase<br>Free laccase+ABTS<br>Lac+ABTS@ZIF-8-Fe	Bisphenol A	12 h	-<br>32.3%<br>36.6%	23.6% at the 3rd cycle

2. Supporting Figures

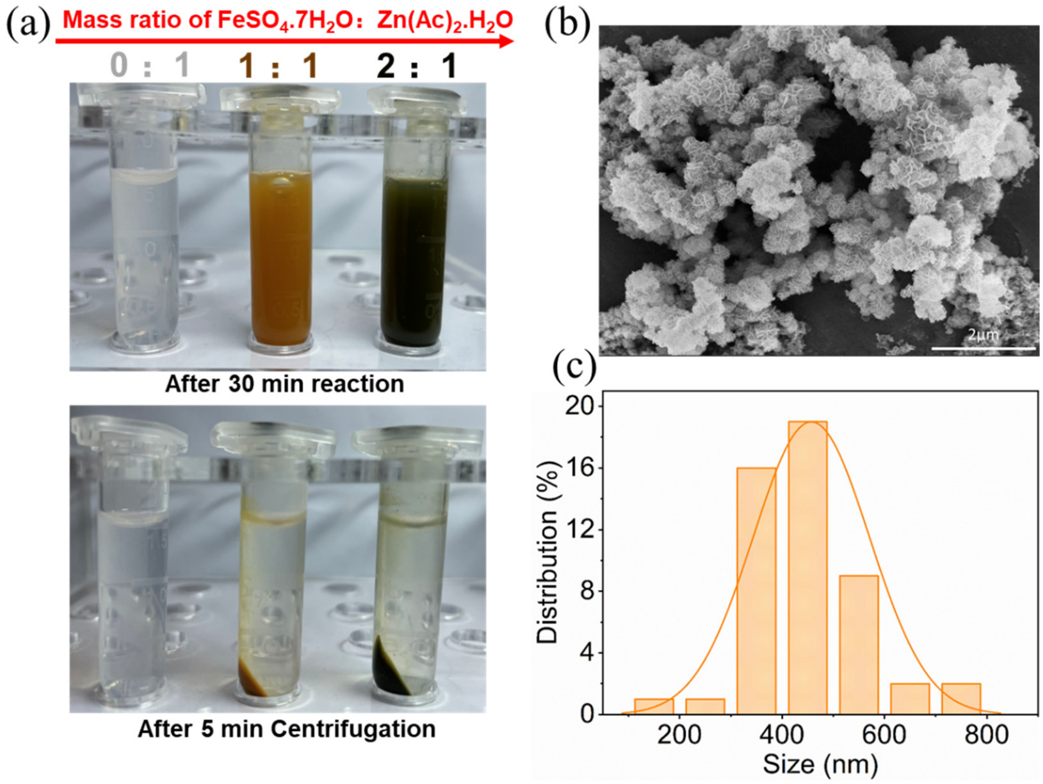

Figure S1. (**a**) Images of synthesis (top) and collection (bottom) processes of the immobilized laccase prepared at different mass ratios of $FeSO_4.7H_2O$ to Zn $(Ac)_2.H_2O$. (**b**) Low-resolution SEM image of Lac@ZIF-8-Fe prepared at a mass ratio of 1 : 1. (**c**) Size distribution histograms of the Lac@ZIF-8-Fe prepared at a mass ratio of 1 : 1.

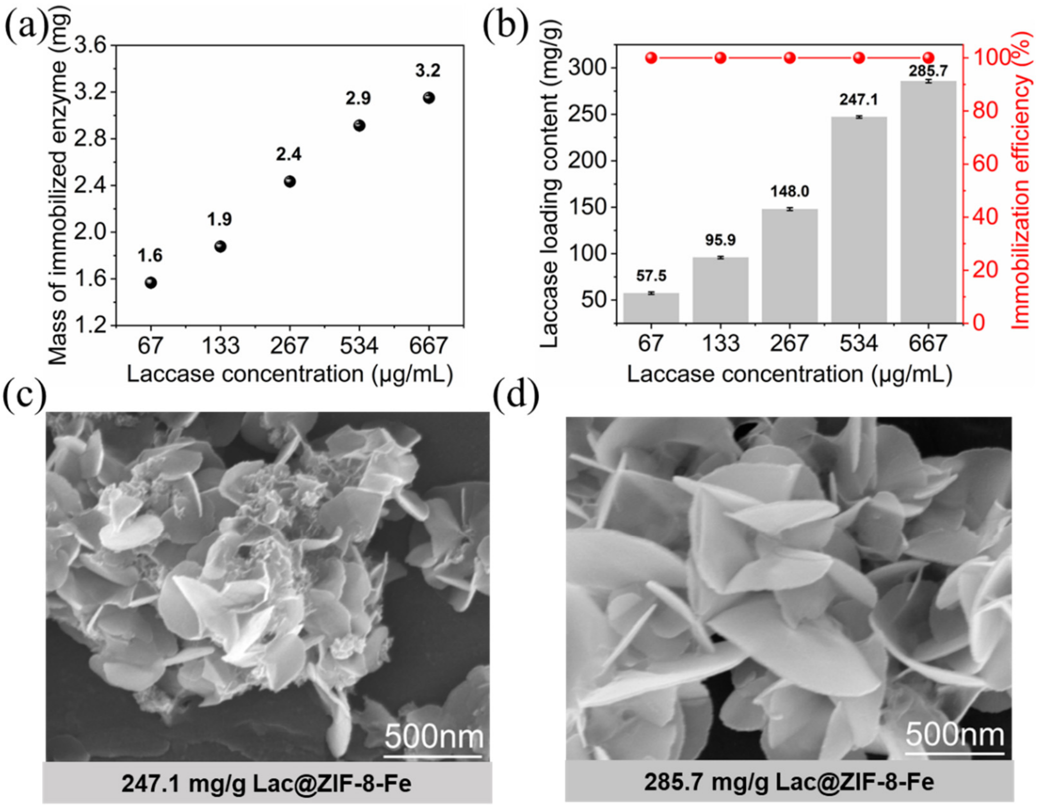

Figure S2. (**a, b**) Effect of laccase concentration on the mass of the immobilized enzyme preparations, laccase loading density, and immobilization efficiency. The total volume in preparation was 1.5 mL. (**c, d**) SEM images of immobilized laccase with enzyme densities of 247.1 (left) and 285.7 mg/g (right).

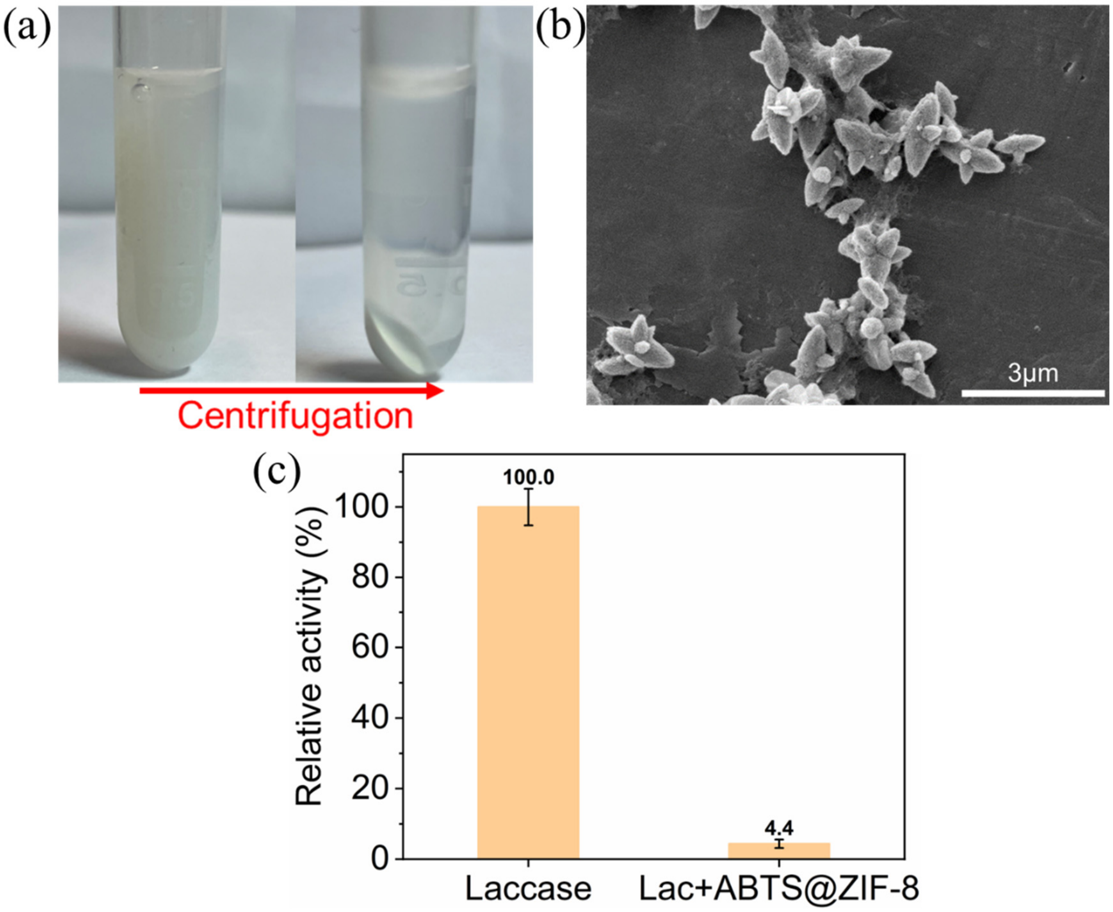

Figure S3. (**a**) Image of the co-immobilized laccase and ABTS into ZIF-8 (Lac+ABTS@ZIF-8). Synthesis condition: Laccase, 66.7 μg/mL; ABTS, 0.67 mg/mL; 2-Hmim, 7.97 mg/mL; Zn $(CH_3COO)_2{\cdot}2H_2O$, 2.67 mg/mL; reaction time, 30 min. The total volume in preparation was 1.5 mL (**b**) SEM image of the synthesized Lac+ABTS@ZIF-8. (**c**) The relative activity of Lac+ABTS@ZIF-8.

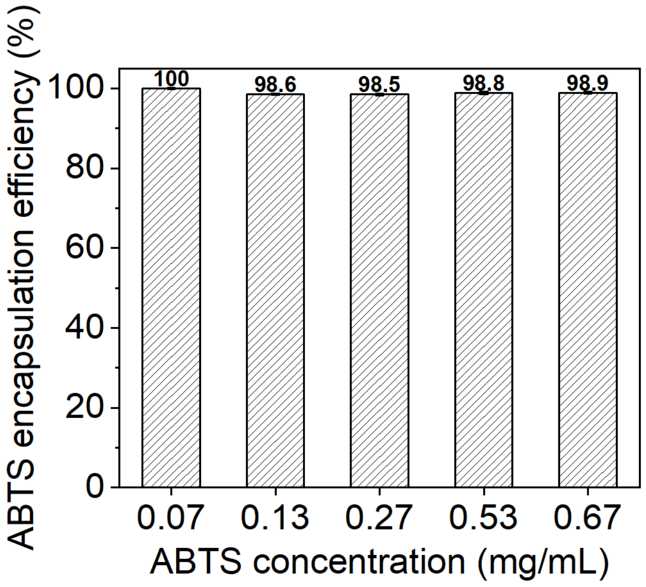

Figure S4. Effect of ABTS concentration on its encapsulation efficiency.

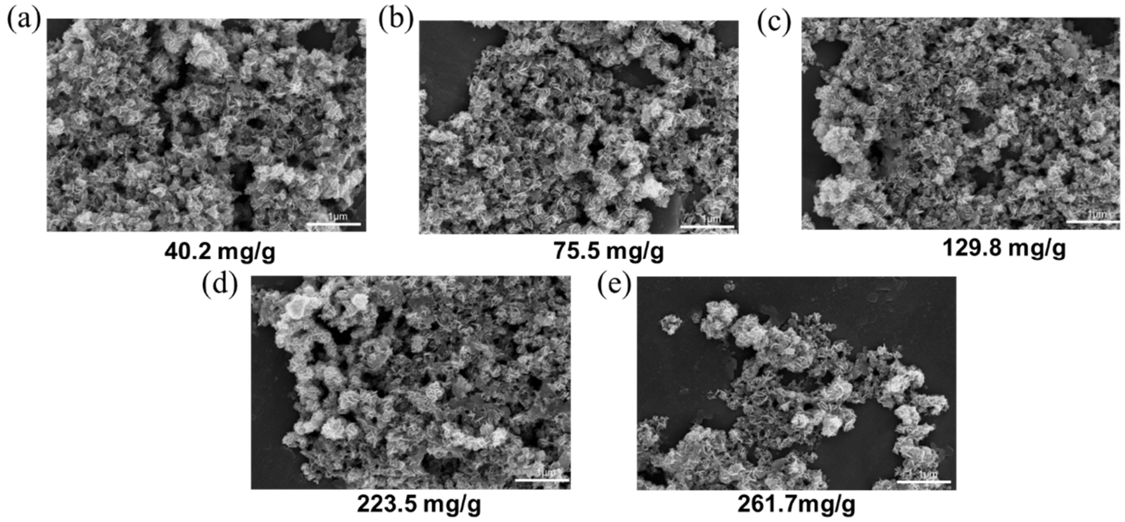

Figure S5. (**a-e**) SEM images of the as-prepared Lac+ABTS@ZIF-8-Fe with different ABTS loading densities denoted below the images (40.2 to 261.7 mg/g).

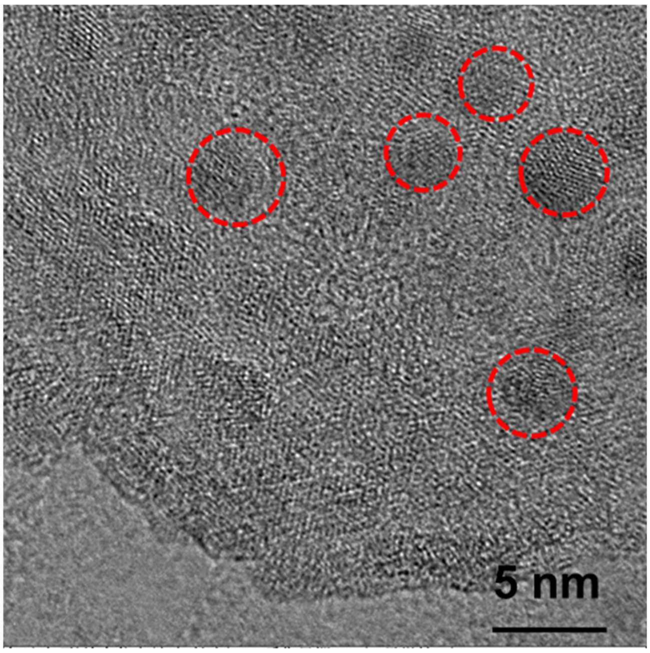

Figure S6. High-resolution TEM image of a nanosheet of Lac@ZIF-8-Fe.

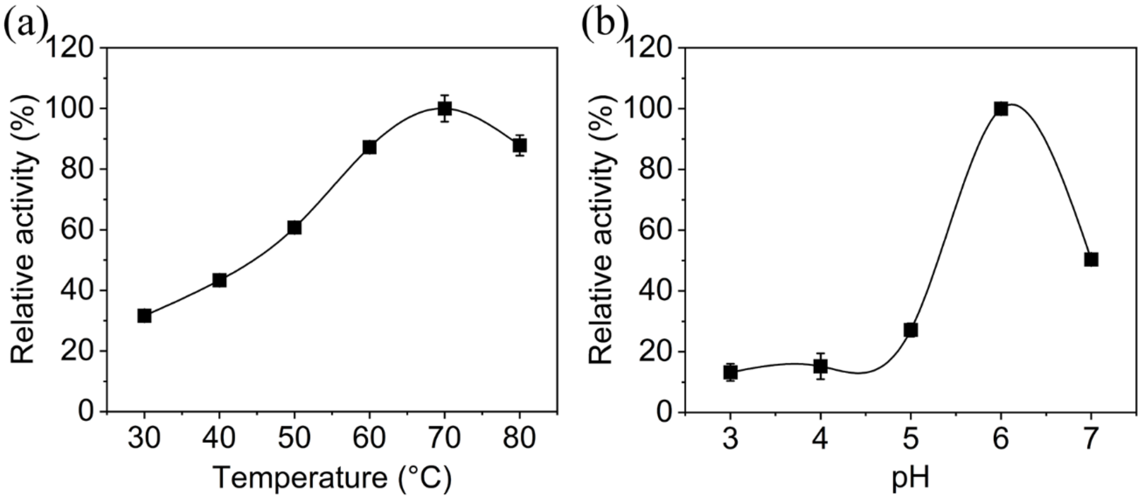

Figure S7. Effects of (**a**) temperature (at pH 6.0) and (**b**) pH (at 50°C) on free laccase for BPA removal. BPA concentration, 5 mg/L; enzyme concentration, 10 μg/mL; reaction time, 4 h.

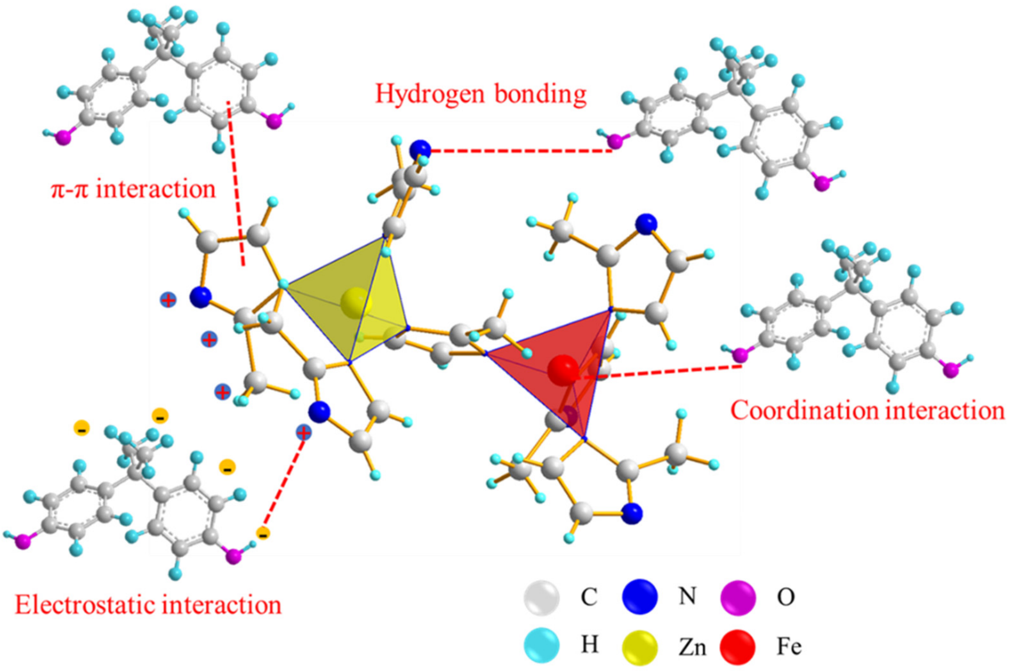

Figure S8. Possible mechanism for BPA adsorption onto ZIF-8-Fe.

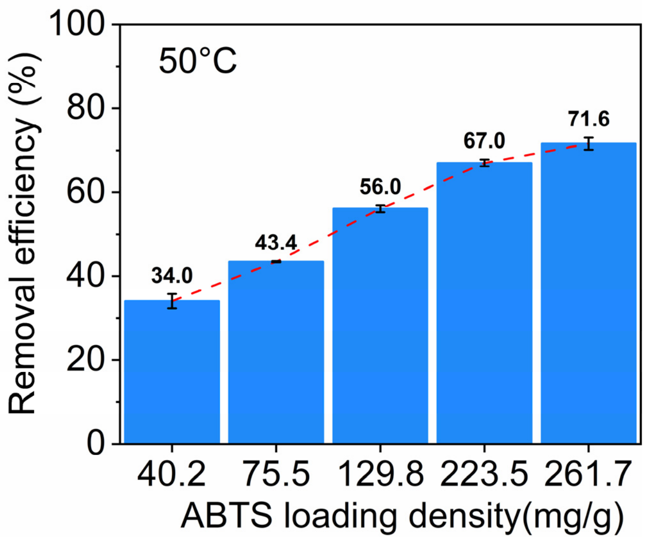

Figure S9. Effect of ABTS loading density of Lac+ABTS@ZIF-8-Fe on BPA removal efficiency at 50℃. BPA concentration, 5 mg/mL; enzyme concentration, 10 μg/mL; reaction time, 4 h.

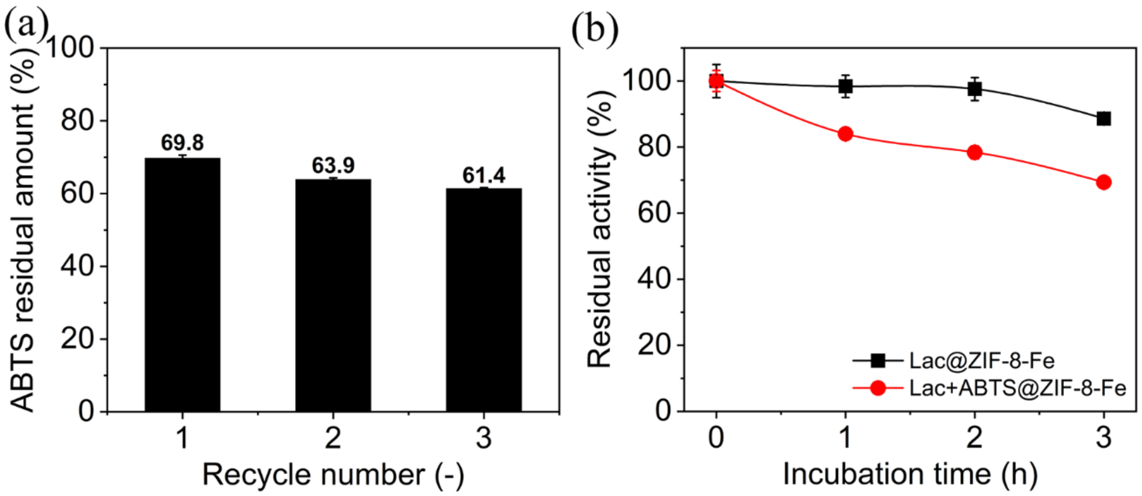

Figure S10. (**a**) ABTS residual amount of Lac+ABTS@ZIF-8-Fe after each cycle during successive MG removal. (**b**) Thermal stability of Lac@ZIF-8-Fe and Lac+ABTS@ZIF-8-Fe at 50°C with 2 mM ABTS as substrate.
